# Self-Collected versus Healthcare Worker-Collected Swabs in the Diagnosis of Severe Acute Respiratory Syndrome Coronavirus 2

**DOI:** 10.3390/diagnostics10090678

**Published:** 2020-09-09

**Authors:** Johan H. Therchilsen, Christian von Buchwald, Anders Koch, Susanne Dam Nielsen, Daniel B. Rasmussen, Rebekka Faber Thudium, Nikolai S. Kirkby, Daniel E. T. Raaschou-Pedersen, Johan S. Bundgaard, Kasper Iversen, Henning Bundgaard, Tobias Todsen

**Affiliations:** 1 Department of Otorhinolaryngology, Head and Neck Surgery and Audiology, Rigshospitalet, Copenhagen University Hospital, 2100 Copenhagen, Denmark; christian.von.buchwald@regionh.dk (C.v.B.); tobiastodsen@gmail.com (T.T.); 2 Department of Infectious Diseases, Rigshospitalet, Copenhagen University Hospital, 2100 Copenhagen, Denmark; Anders.Koch@regionh.dk (A.K.); susanne.dam.poulsen@regionh.dk (S.D.N.); daniel.braeuner.rasmussen@regionh.dk (D.B.R.); rebekka.faber.thudium@regionh.dk (R.F.T.); 3 Department of Clinical Microbiology, Rigshospitalet, Copenhagen University Hospital, 2100 Copenhagen, Denmark; nikolai.kirkby@regionh.dk; 4 Department of Cardiology, Rigshospitalet, Copenhagen University Hospital, 2100 Copenhagen, Denmark; daniel.raaschou@gmail.com (D.E.T.R.-P.); johan.bundgaard@gmail.com (J.S.B.); Henning.bundgaar@regionh.dk (H.B.); 5 Department of Cardiology, Copenhagen University Hospital Herlev, 2730 Herlev, Denmark; kasper.iversen@regionh.dk; 6 Copenhagen Academy for Medical Education and Simulation, Rigshospitalet, Copenhagen University Hospital, 2100 Copenhagen, Denmark

**Keywords:** COVID-19, COVID-19 diagnostic testing, severe acute respiratory syndrome coronavirus 2

## Abstract

The aim of this study was to compare the sensitivity of self-collected versus healthcare worker (HCW)-collected swabs for Severe Acute Respiratory Syndrome Coronavirus 2 (SARS-CoV-2) testing. Symptomatic individuals referred for SARS-CoV-2 testing were invited to provide mobile-phone video-instructed self-collected oropharyngeal and nasal samples followed by a HCW-collected oropharyngeal sample. All samples were sent for analysis to the same microbiology laboratory, and the number of SARS-CoV-2-positive participants in the two tests was compared. A total of 109 participants were included, and 19 participants had SARS-CoV-2-positive results. The diagnostic sensitivity of the self-collected and HCW-collected swabs was 84.2% and 89.5%, respectively, with an acceptable agreement, Cohens kappa 0.82, *p* < 0.001. Further, results from a questionnaire answered by the participants found that loss of smell as a self-reported symptom was a strong predictor for a SARS-CoV-2-positive test. In conclusion, we found that self-collected oropharyngeal and nasal swabs for SARS-CoV-2 testing can be reliable compared to HCW-collected oropharyngeal samples.

## 1. Introduction

A comprehensive Severe Acute Respiratory Syndrome Coronavirus 2 (SARS-CoV-2) testing strategy is recommended to quickly identify new cases and suppress local outbreaks during the current coronavirus disease 2019 (COVID-19) pandemic. The WHO recommends a collecting technique for upper respiratory samples to obtain oropharyngeal and nasopharyngeal samples, which is performed by a healthcare worker (HCW) [1]. This approach has a high economic burden, reduces the number of available HCWs for other tasks, increases depletion of personal protective equipment, and exposes the HCWs to the risk of infection. Therefore, alternative methods for the collection of specimens for viral analyses are desired [2]. Self-collected swabs may be a low-cost alternative to HCW-collected samples for SARS-CoV-2 testing, as supported by earlier studies which found that self-collected samples are reliable in testing for influenza virus [3,4,5,6]. Self-testing is now accepted as an initial diagnostic testing method for SARS-CoV-2 by the National Health Service (NHS, United Kingdom) [7] and the Centers for Disease Control and Prevention (CDC, USA) [8], even though data on the diagnostic accuracy of self-collected swabs for SARS-CoV-2 testing are sparse [9,10,11,12]. The aim of this study was to explore the correlation and diagnostic sensitivity of a simple low-cost technique for self-collected samples as an alternative to the more burdensome method based on HCW-collected samples in the diagnosis of SARS-CoV-2 in symptomatic individuals.

## 2. Methods

We performed a cross-sectional study to compare the diagnostic results when both self-collected and HCW-collected swabs for SARS-CoV-2 testing were obtained from the same individuals with symptoms indicative of COVID-19.

### 2.1. Setting and Eligibility Criteria

Eligible study participants were persons referred to the COVID-19 outpatient testing facility at Copenhagen University Hospital Rigshospitalet, Denmark. Persons were referred by a general practitioner because of mild symptoms compatible with COVID-19, for screening prior to an outpatient appointment, or in relation to a planned hospital admission (e.g., surgery at the hospital). Further, symptomatic HCWs employed at the hospital were also referred for COVID-19 testing at the facility.

**Figure 1:**
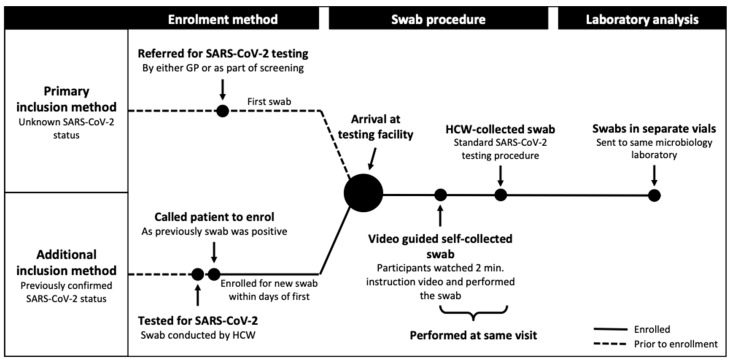
Study flowchart. SARS-CoV-2, severe acute respiratory syndrome coronavirus 2, GP, general practitioner, HCW, healthcare worker.

In the period 5 May–1 July 2020, persons above 18 years of age attending the testing facility with symptoms of upper respiratory tract infection were asked to participate in the study. There were no exclusion criteria. In addition, to increase the number of SARS-CoV-2-positive patients in the study, we also invited already confirmed SARS-CoV-2-positive patients to participate in the study. These patients received a phone call informing them of their SARS-CoV-2-positive status from a previous test. If they agreed, a new appointment for SARS-CoV-2 testing—as a part of the study—was scheduled, i.e., within a few days following their initial SARS-CoV-2-positive sample, see Figure 1.

### 2.2. Interventions

**Figure 2:**
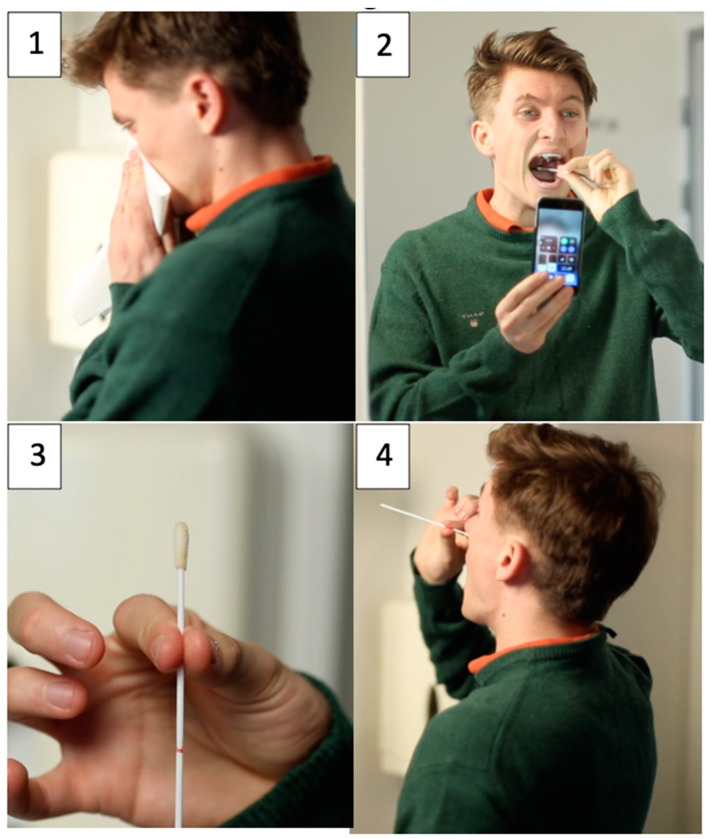
Screenshots from the Supplementary Video S1 demonstrating how to perform self-collection of oropharyngeal and lower nasal samples with the use of a single swab for SARS-CoV-2 testing. Foto 1 shows the patient examining which nostril to use for collection the sample. Foto 2 shows collection of oropharyngeal sample. Foto 3 shows depth of insertion to collect middle turbinate sample. Foto 4 shows the patient performing middle turbinate sample. For more information please consult with Figure A1.

At arrival at the testing facility, persons referred for testing were informed about the study verbally and in writing. After giving informed consent, those volunteering for the study provided a self-collected sample and a HCW-collected sample. Self-collection of samples was done in a separate room at the COVID-19 testing facility, where the study persons received written and Supplementary Video S1 instructions. The two-minute long instructional Supplementary Video S1 was produced specifically for the present study and demonstrated how to perform self-collection of an oropharyngeal sample, using a mirror and the light from the person’s mobile phone, and of a nasal (mid-turbinate) sample, using the same rigid shafted swab, with a hydroflocked tip (Zymo Collection Swab, Zymo Research, Irvine, CA, USA), as shown in Figure 2.

**Figure A1:**
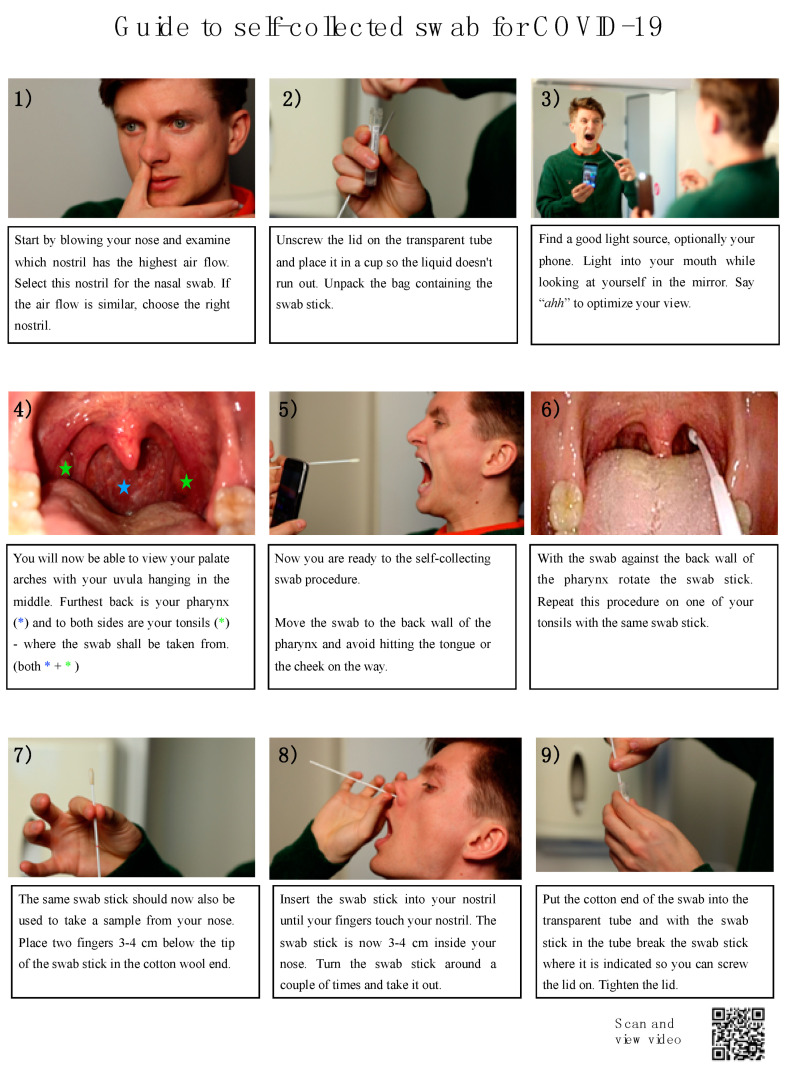
Written Instructions.

**Figure A2:**
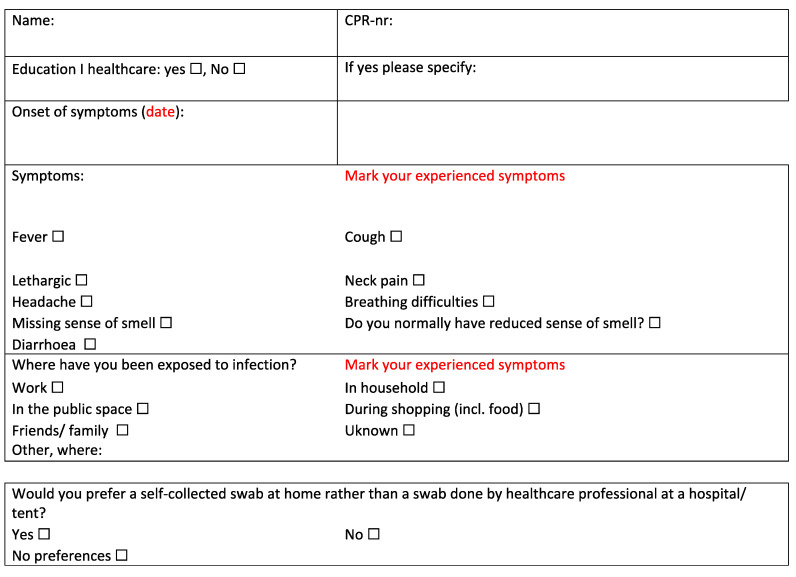
Questionnaire to the participants.

For the oropharyngeal sample, both the posterior wall of the oropharynx and one palatine tonsil had to be sampled for the sampling technique to be considered correct; for the nasal sample and correct mid-turbinate sampling, the swab had to be introduced for about 3 cm or till resistance was met. The participants watched the Supplementary Video S1 on their own mobile phone and thereafter performed the self-sampling directed by the written pictorial guide summarizing the steps in the procedure (see Figure A1). Without providing any guidance, a HCW was present in the room observing and registering possible sampling errors while the participants performed the self-collecting swab procedure. Afterwards, a trained medical student collected an oropharyngeal sample from the opposite tonsil and posterior wall of the oropharynx, according to the standard procedure to obtain HCW-collected samples for SARS-CoV-2 testing in Denmark. The self-collected and HCW-collected swabs were placed in individually labelled vials (eSwab Collection & Transport System, Copan Italia SpA, Brescia, Italy) and stored at 4 °C until transportation to the Department of Clinical Microbiology, Rigshospitalet, which performed all analyses of SARS-CoV-2 samples at the hospital. At the completion of both swab collections, the participants were asked to complete a questionnaire regarding their symptoms and an evaluation of the sampling procedures (see Figure A2).

### 2.3. SARS-CoV-2 Real-Time Reverse-Transcription Polymerase Chain Reaction Testing

The swabs were processed as routine samples by clinical laboratory technicians blinded to the sampling method. Samples were analyzed using the real-time reverse-transcription polymerase chain reaction (rRT-PCR) assay, by using either the SARS-CoV-2 real-time RT-PCR test on the Cobas 6800 system (Roche, Basel, Switzerland) or the RealStar^®^ SARS-CoV-2 RT-PCR Kit (Altona, Hamburg, Germany). In brief: the nucleic acids in the patient sample were extracted together with an internal RNA control using magnetic silica particles and transferred to a specific RT-PCR assay, targeting two separate gene segments.

### 2.4. Statistical Analysis

A true positive test result was defined as a SARS-CoV-2-positive result from either the self- or the healthcare-collected sample. Intertest agreement between the self-collected and the HCW-collected swabs was calculated using Cohen’s kappa (k). A k > 0.80 was considered an acceptable intertest agreement [13]. The sensitivity of each test was calculated as the number of positive test/total number of true positive patients for both the self- and the HCW-collected samples. Differences in group characteristics were compared with the chi-square test for categorical variables and the Student’s *t*-test for continuous variables; *p*-values were Bonferroni-corrected because of multiple statistical testing. The statistical analysis was performed using a statistical software package (PASW, version 26.0; SPSS Inc, Chicago, IL, USA), and two-sided significance levels of 0.05 were used for all analyses.

### 2.5. Ethics and Data Management

Ethical approval was granted in the form of an exemption letter from the regional ethical committee of the Capital Region of Denmark (protocol no. H-20027981, approved on 24 April 2020), and the Danish Data Protection Agency approved the management of patient-sensitive information during the study (record number: P-2020-467). All participants gave verbal and written informed consent prior to enrolment.

## 3. Results

A total of 109 participants were included in the study. Demographics of the participants and their self-recorded symptoms are shown in Table 1.

Among the 109 participants, 19 patients had SARS-CoV-2-positive results from self-collected samples, HCW-collected samples, or both. The proportion of SARS-CoV-2-positive samples was 16/109 (14.7%) for the self-collected samples in comparison to 17/109 (15.6%) for the HCW-collected samples, as shown in Table 2. Acceptable agreement between self-collected and HCW-collected swabs was found, Cohens kappa 0.82, *p* < 0.001, without any significant difference in diagnostic sensitivity for the self-collected and HCW-collected samples, corresponding to 84.2% and 89.5%, respectively, *p* = 0.81. However, of the 19 positive samples, only 14 (74%) were found positive by both tests. Combining the self-collected samples to the HCW-collected samples added two more SARS-CoV-2-positive cases out of the 109 included participants, who would otherwise have been tested as false negative using only the HCW-collected samples.

As to the preference of the swab technique, 47/109 (43.1%) of the participants preferred sample self-collection, 29/109 (26.6%) preferred collection by HCWs, and 33/109 (30.3%) did not have any preference. An error in the sampling technique by a participant was registered for 16/109 (14.7%) of the self-collected oropharyngeal samples, 18/109 (16.5%) for nasal samples; 4/109 (3.7%) of the participants made a double error. Six sampling errors were observed in the 19 SARS-CoV-2-positive patients, including a double sampling error, and in 1/3 of the false negative participants, a sampling error for the middle turbinate sample was observed. Discordant results favoring HCW collection regarded primarily patients 20–25 days post-symptom onset. If inclusion of patients were limited to two weeks post-symptom onset, then 14/14 self-collected vs. 13/14 HCW-collected samples would have been positive for SARS-CoV-2. A subgroup analysis found that samples from participants with a healthcare education detected SARS-CoV-2 in 5/6 (83%) of the true positive cases; a similar detection rate was found for the participants without a healthcare education 11/13 (85%). 

Loss of smell was the only self-recorded symptom that differed significantly between SARS-CoV-2-positive and SARS-CoV-2-negative participants (53% versus 9%, *p* < 0.001) (Table 1).

## 4. Discussion

We found that self-collection of sample swabs for SARS-CoV-2 testing is a well-tolerated diagnostic method with a sensitivity almost equivalent to that of collection of oropharyngeal samples by HCWs. A very good and significant intertest correlation was found between the diagnostic outcomes of self-collected and HCW-collected samples, although we found a slightly lower sensitivity for the self-collected samples compared with the HCW-collected ones. The sensitivity of 84.2% of the self-collected samples is comparable to values found in other studies [9,10,11] and slightly higher than that determined in a study from Washington, USA [12]. However, in the latter study, there was a delay between the diagnostic tests and the shipping of the self-collected samples at room temperature from participants’ homes, which could explain the observed difference [12]. A strength of our study is that the self- and HCW-collected samples were obtained at the same time, which decreased the risk of change of viral load between the tests. Further, all of the 218 (109 × 2) samples were stored in the same type of transport medium and at the same temperature and analyzed at the same laboratory, which increases the internal validity of our test results. We used a simple self-collection sampling technique in this study, whereby all participants were guided by a mobile phone video-instruction, and no HCW were needed for guidance. This could therefore increase the applicability of the method using a single swab and a vial for self-testing.

Interestingly, the loss of smell was the only self-reported symptom for which a difference was seen between SARS-CoV-2-positive and -negative participants. Approximately half of the SARS-COV-2-positive participants reported a loss of smell compared to 9% of the SARS-COV-2-negative participants, a result that is highly significant even after a Bonferroni correction for multiple statistical testing. These findings are comparable with those of other studies describing olfactory loss associated with SARS-CoV-2 infection [14,15]. Therefore, we recommend heightened awareness when patients describe a sudden loss of smell, and it should be considered whether to offer them a test for SARS-CoV-2.

The findings of the present study should be interpreted in accordance to the following limitations.

We do not know the true SARS-CoV-2 infection status in our participants, as our results are dependent on a positive HCW-collected and/or self-collected swab. We can therefore expect that some of the participants will be false-negative, and a lower sensitivity of both tests may be expected. Further, the HCW-collected and self-collected swabs were not performed in the same way, as the self-collection technique also included the acquisition of a nasal sample in addition to the oropharyngeal sample. However, as the study aim was to compare a new self-testing technique with the recommended standard technique for SARS-CoV-2 testing in Denmark (HCW-collected oropharyngeal sample), we chose this method. The small sample size of our study was without sufficient power to conduct a non-inferiority statistical comparison of the sensitivity between HCW-collected and self-collected samples for SARS-CoV-2 testing. A priori, we did not expect self-collected samples to be as sensitive as HCW-collected, and we were surprised by the almost equivalent sensitivity of the self-collection- and HCW-collection-based SARS-CoV-2 testing methods found in our study. These findings may be the result of combined oropharyngeal and nasal sampling for the self-collected samples that were compared to HCW-collected oropharyngeal swabs. In our study, we found two additional SARS-CoV-2-positive patients—corresponding to an increase in sensitivity of 10%—by adding self-collected oropharyngeal and nasal samples to HCW-collected oropharyngeal swabs. Assuming a similar sensitivity for the self- and the HCW-collected samples, this emphasizes the importance of nasal samples in SARS-CoV-2 testing. These findings are also in accordance with a recent study that found a higher concentration of SARS-CoV-2 RNA in nasopharyngeal samples than in oropharyngeal ones in SARS-CoV-2-positive patients [16]. Another limitation is the presence of a high proportion of health professionals among the participants, which may impact on the generalizability of our results. A lower sensitivity of the self-collected swabs could be expected when the technique is introduced in the general community, with older and fewer healthcare-educated patients. However, we found no difference in the proportion of false-negative test results between participants with or without a healthcare education. Another factor which might impact the sensitivity of the test method were it to be performed without a HCW’s supervision is the patients’ personal situation. Some patients might have a social or economic interest or a fear of a positive result.

In conclusion, we found an acceptable sensitivity for swab samples collected from the nasal and oropharyngeal cavities for SARS-CoV-2 testing by patients solely guided by a written pictorial guide and Supplementary Video S1 instructions on their mobile phone. The SARS-CoV-2 self-test described here is therefore a reliable testing method with a low false-negative rate compared to the technique based on HCW-collected swabs and might be used in community testing or settings where HCW time and personal protective equipment need to be economized. Future studies should explore the diagnostic accuracy and cost-effectiveness of this method when implemented in a larger and more heterogenous cohort of patients tested for COVID-19.

## Figures and Tables

**Table 1:** Clinical characteristics and self-recorded symptoms of the study participants.

	All Participants	SARS-CoV-2 Negative	SARS-CoV-2 Positive	*p* Value (Chi-Square)
**Clinical characteristics**				
Number of participants	109	90	19	
Sex, female–n (%)	76 (70%)	65 (72%)	11 (58%)	0.22
Mean age—years mean/median (SD)	39 (13)	40.4 (13)	32.6 (5.9)	0.33
Healthcare education n (%)	26 (25%)	21 (23%)	6 (32%)	0.45
Median days since first symptoms mean/median—(Range) IQR	3.0 (0–65)	3.0 (0–65)	7.0 (2–25)	0.10
**Self-recorded Symptoms, n (%)**				
Fever	41 (38)	32 (36)	9 (47)	0.35
Cough	54 (50)	44 (49)	10 (53)	0.80
Lethargy	55 (50)	43 (48)	12 (63)	0.24
Throat pain	59 (54)	51 (57)	8 (42)	0.23
Headache	62 (57)	48 (53)	14 (74)	0.11
Respiratory problems	14 (13)	12 (13)	2 (11)	0.11
Loss of smell	18 (17)	8 (9)	10 (53)	<0.001 *
Diarrhea	14 (13)	11 (12)	3 (16)	0.67

* *p*-value after Bonferroni correction.

**Table 2:** Samples positive for SARS-CoV-2 by sampling method.

Participant (SARS-CoV-2 True Positive)	Time from Self-Reported Symptom Onset	Healthcare Worker-Collected Oropharyngeal Swab	Self-Collected Middle Turbinate/Oropharyngeal Swab
1	8	Positive	Positive
2	25	Positive	Negative
3	12	Positive	Positive
4	3	Positive	Positive
5	2	Positive	Positive
6	7	Positive	Positive
7	9	Positive	Positive
8	2	Positive	Positive
9	5	Positive	Positive
10	5	Negative	Positive
11	25	Negative	Positive
12	4	Positive	Positive
13	3	Positive	Positive
14	10	Positive	Positive
15	17	Positive	Positive
16	6	Positive	Positive
17	20	Positive	Negative
18	6	Positive	Positive
19	23	Positive	Negative

## Supplementary Materials

The instruction Supplementary Video S1 was constructed specifically for this study. https://drive.google.com/file/d/1ch4At6pvYThB5qKz32PFUJ69aOUur2Wg/view?usp=drive_web.
